# Reorganization of the Connectivity between Elementary Functions – A Model Relating Conscious States to Neural Connections

**DOI:** 10.3389/fpsyg.2017.00625

**Published:** 2017-04-20

**Authors:** Jesper Mogensen, Morten Overgaard

**Affiliations:** ^1^The Unit for Cognitive Neuroscience, Department of Psychology, University of CopenhagenCopenhagen, Denmark; ^2^Cognitive Neuroscience Research Unit, Center of Functionally Integrative Neuroscience, MindLab, Aarhus UniversityAarhus, Denmark

**Keywords:** neural correlate of consciousness (NCC), consciousness, neural connections, cognition, mental states, neural states, computational states, integrative models

## Abstract

In the present paper it is argued that the “neural correlate of consciousness” (NCC) does not appear to be a separate “module” – but an aspect of information processing within the neural substrate of various cognitive processes. Consequently, NCC can only be addressed adequately within frameworks that model the general relationship between neural processes and mental states – and take into account the dynamic connectivity of the brain. We presently offer the REFGEN (general reorganization of elementary functions) model as such a framework. This model builds upon and expands the REF (reorganization of elementary functions) and REFCON (of elementary functions and consciousness) models. All three models integrate the relationship between the neural and mental layers of description via the construction of an intermediate level dealing with computational states. The importance of experience based organization of neural and cognitive processes is stressed. The models assume that the mechanisms of consciousness are in principle the same as the basic mechanisms of all aspects of cognition – when information is processed to a sufficiently “high level” it becomes available to conscious experience. The NCC is within the REFGEN model seen as aspects of the dynamic and experience driven reorganizations of the synaptic connectivity between the neurocognitive “building blocks” of the model – the elementary functions.

## Relating Conscious States to Neural Connections

The idea of a “NCC” has been around for decades and was probably first used in print by Crick and Koch (1990). The search for a NCC has often focused on anatomically defined brain structures as candidates for such a role – e.g., the prefrontal cortex and parts of the primary sensory areas (for review see Koch et al., 2016). Other models emphasize types of processes rather than specific structures (e.g., Tononi, 2004; Lamme, 2010). Elsewhere (Overgaard and Mogensen, 2011) we have stressed some of the issues to be considered when searching for a NCC – and later emphasized models with a primary focus on the ways in which information is integrated in the processing of the brain (e.g., Overgaard and Mogensen, 2014; Overgaard and Mogensen, 2017).

The patterns of neural information processing crucially depend on the pattern of neural connections. Consequently, a better understanding of NCC may depend on (a) an improved mapping of the connectivity of the brain and (b) methods and models allowing the association between these patterns of connectivity and conscious processes, respectively, to be understood. While significant progress is being made regarding the first of these issues, there has been more modest progress regarding the second. Below, we suggest a framework integrating studies of the connectivity of the brain and the search for a NCC.

Evidence from various sources advocates against an absolute dichotomy between conscious and non-conscious states (for reviews see, e.g., Overgaard and Mogensen, 2014, 2015). Considering the available data, we have proposed that when information within various cognitive domains is processed to a sufficiently “high level” it becomes available to conscious experience (e.g., Overgaard and Mogensen, 2014, 2015, 2017). Thus, we do not see consciousness as a separate “module” or faculty – but rather as a – to varying degrees present – aspect of other cognitive processes. Consequently, we believe that in order to account for a NCC, a framework needs to model the more general relationship between neural processes and mental states – and to address consciousness and NCC within general neurocognitive models integrating in principle all aspects of cognition.

Regarding the process of relating neural circuits and connections to behavior or mental states, Carandini (2012) has argued that the explanatory gap between these two levels is too wide to obtain a reasonable link. He insists on the need for an “intermediate level.” And that computations based on neural processing provide a language for theories of behavior so that computations form such an intermediate level. These arguments resemble the classic levels suggested by Marr and Poggio (1977) and Marr (1982). Marr proposed three levels of analysis: (1) a computational level, (2) an algorithmic level, and (3) an implementation level. There may be disagreements regarding how to characterize and term the levels, but these and other models converge on the idea that one or another type of algorithmic intermediate level is needed in order to bridge the neural and mental/behavioral levels of analysis.

Extensive human and not the least animal model based studies (e.g., Wörtwein et al., 1995; Mogensen et al., 2002, 2004, 2005, 2007; Malá et al., 2015; Ishkhanyan et al., 2017) point to two important principles. (A) A surface phenomenon such as task performance or conscious representations may before and after injury, respectively, be mediated by dissimilar neural substrates and computational processes – although the surface phenomena superficially viewed are similar (e.g., demonstrating task performance of similar proficiency). Furthermore, (B) contributions to task mediation provided by individual substructures appear to be of a “modular” nature – contributing information processing which is not task-specific – or for that matter specific to any of the cognitive domains – but rather contributes the same type of analysis within a multitude of different contexts/tasks (Mogensen and Malá, 2009; Mogensen, 2011a,c, 2012a,b, 2014, 2015).

**FIGURE 1:**
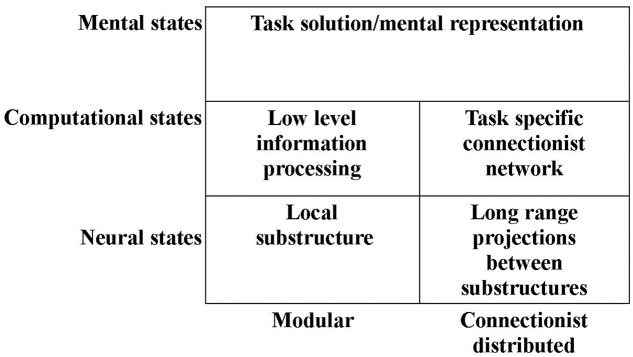
**Illustration of the subdivision of the computational and neural levels into modular and connectionist distributed subdivisions (see text for details)**.

Thus, it may be tempting to operate with not only one computational level, but two such levels: One level at which the computations are those provided by the local networks within substructures of the brain. And a “higher” level of hypercomputations – a level at which mental representations are mediated by a connectionist network combining the specific modules of the previous level (see **Figure 1**). We have developed such a novel approach to the construction of a computational level and an alternative understanding of modularity (e.g., Mogensen and Malá, 2009; Mogensen, 2011a,c, 2012a,b, 2014, 2015; Overgaard and Mogensen, 2011, 2014, 2015).

## A New Understanding of Modularity

Although this approach resembles models of “modular” brain organization (e.g., Pinker, 1999; Fodor, 2000; Barrett and Kurzban, 2006) it differs in a number of important ways from what is normally associated with modularity. In fact, our approach may explain what underlies the modularity suggested by many cognitive neuroscience findings. It may also suggest an alternative approach to delineation and selection of nodes for connectome research (e.g., Zalesky et al., 2010).

(I) The computations behind any task solution, conscious representation or other phenomenon at the mental level is related to activity within a distributed network combining a multitude of individual modules. The fixed computations of individual modules are never the background of mental phenomena in isolation. Thus, modularity is embedded within highly dynamic computational networks in which backpropagation mechanisms adjust the computations according to constantly changing environmental demands (e.g., McClelland et al., 1986; Rumelhart and McClelland, 1986; McLeod et al., 1998; Mogensen, 2012b; Rogers and McClelland, 2014).
(II) Approaches focusing on which cognitive domains are impaired after lesions within a particular part of the brain have apparently identified neural and computational “modules.” The Broca area, for instance, is mostly seen as a “module” for linguistic processes. However, the refined analysis of not only whether but how the computations and mental phenomena are modified by lesions of such brain regions negate the role as a single module exclusively associated with one higher level cognitive domain (e.g., Mogensen, 2011a,c, 2012b, 2014, 2015; Overgaard and Mogensen, 2011). Rather, apparently domain-specific brain structures should be seen as collections of computationally specific modules with neural substrates far smaller than for instance a traditional cortical area or nucleus of a subcortical structure. In case of the Broca area, these subdivisions are significantly smaller than the subdivisions described by for instance Anwander et al. (2007). While such neural and computational modules may most frequently be involved in one of the traditionally defined cognitive domains (e.g., expressive language), the modules are not specific to that domain. And they may under different circumstances be involved in apparently different “functions.” For instance in musicians (but not in other subjects) the Broca area is involved in mediation of mental rotation (Sluming et al., 2007).
(III) We do not challenge the existence of individual modules conducting fixed computations. But the modules, we believe, are units within distributed connectionist networks. And each module thus contributes to many of the traditional cognitive domains. Consequently, one should not expect the higher order cognitive domains to show the kind of mutual isolation and separation of information processing assumed in many modular theories (e.g., Pinker, 1999; Fodor, 2000). Also, entities such as “the language faculty” of Chomsky (1965, 1975, 1986, 2000) and Hauser et al. (2002) are in this context not seen as neither isolated nor unitary systems. It seems more promising to look for lower level information processing units which are common to several higher level cognitive faculties. An example is the linguistic theory of Boye and Harder (2012) where aspects of grammar are suggested to be processed by the same computations as those processing information regarding “background”/“foreground” in perception – an assumption already supported by data within linguistics (e.g., Rosenberg et al., 1985) and fMRI-based studies (Kristensen et al., 2013). Also, rehabilitative cognitive training after brain injury may prove to be of therapeutic value even when apparently unrelated to the impaired cognitive domain (e.g., Wilms and Mogensen, 2011).

These principles fit well with Friston’s (1997) concepts of functional integration and functional segregation. For instance, the Broca area is not conceptualized as a functional entity to which a particular higher level function is “localized” but contains a multitude of separate lower level functional entities. This constitutes a functional segregation closely related to the one of Friston (1997). The computational and neural networks behind higher level cognitive functions – networks presumably sharing at least some basic mechanisms – are manifestations of a functional integration. The Integrated Information Theory (IIT) (e.g., Tononi et al., 2016) represents another approach based on the interaction of functional segregation and integration.

Cognitive recovery after brain injury is achieved by a neural and cognitive reorganization leading to a (potentially fully proficient) task solution – which is achieved via neural and cognitive mechanisms dissimilar to those found under normal circumstances (e.g., Mogensen and Malá, 2009; Mogensen, 2011a,c, 2012b, 2014, 2015). Also, the pattern of such a neural and cognitive reorganization depends on the type of cognitive task faced by the injured individual (see above – and Mogensen and Malá, 2009; Mogensen, 2011a,c, 2012b, 2014, 2015).

There is an apparent contradiction between the concepts of “localization” of various “functions” (e.g., Coltheart, 2001; Selnes, 2001; Kringelbach and Rolls, 2004) and functional “recovery” as seen in animal models (see references above) and clinical studies (e.g., Ansaldo and Arguin, 2003; Perani et al., 2003; Baumgaertner et al., 2005; Specht et al., 2009; Szaflarski et al., 2011). The two phenomena have a number of parallels to two of the prominent models of functional organization within the brain: The “modular” theories (e.g., Pinker, 1999; Fodor, 2000; Barrett and Kurzban, 2006), and the models emphasizing distributed, connectionist networks (e.g., McClelland et al., 1986; Rumelhart and McClelland, 1986; McLeod et al., 1998; Rogers and McClelland, 2014). Modularity emphasizes a strict functional localization in which loss of the neural structure mediating that cognitive module leads to a cognitive impairment. On the basis of such a model it is, however, hard to realize how a function may demonstrate a posttraumatic functional recovery. On the other hand, connectionist models make it easier to conceptualize the dynamic network reorganizations seen in posttraumatic cognitive rehabilitation – but can rarely account for the degree and specificity of initial trauma-associated impairments (e.g., Mogensen, 2011a, 2012b).

## The REF Model

Attempting to establish a comprehensive model of the neural and cognitive mechanisms of posttraumatic functional recovery of problem solving, the REF model was constructed (Mogensen and Malá, 2009; Mogensen, 2011a,c, 2012a,b, 2014, 2015). The REF model essentially describes a connectionist network in which, however, the “unit” is not a neutral and functionally “indifferent” “neuron” – but advanced information processing modules called elementary functions (EFs). This model is able to account for both the localization and posttraumatic recovery of functions.

According to the original version of the REF model (Mogensen and Malá, 2009; Mogensen, 2011a,c, 2012a) the surface level of task solution – be it in the form of overt behavior or mental representation – is achieved via two underlying levels: the lower level of the EFs and the level of the Algorithmic Strategies (ASs). EFs perform basic information processing and are localized within restricted subdivisions of neural structures. In contrast, ASs consist of numerous interacting EFs and are distributed in the sense that the neural substrate of an AS includes both the neural substrates of the individual EFs and the neural connections mediating the complicated interaction between these EFs. Thus, an AS is the totality of a given set of EFs and their interactions. A given surface phenomenon is achieved via the computations of a given AS. In a later elaboration of the REF model, the level of algorithmic modules (AMs) has been added (Mogensen, 2012b, 2014, 2015). Like an AS, an AM consists of a number of EFs and a pattern of interconnections between these EFs – computationally constituting a significantly higher level of information processing than what is achieved by an individual EF. AMs, however, differ from ASs by not being able in themselves to mediate a task solution. Rather, AMs are “building blocks” common to a number of ASs – performing a higher level of information processing than the more basic “building blocks,” the EFs.

A given surface phenomenon (e.g., behavioral pattern/task solution) may be achieved via different ASs. Focal brain injury will deprive the individual of a substantial number of EFs and thereby all ASs including those EFs. Thus, injury will lead to behavioral impairments of tasks previously achieved via activation of those ASs (e.g., Mogensen, 2011a, 2012b). Subsequent training will, however, be able to establish novel ASs (utilizing preserved EFs). And potentially the novel ASs will allow a task solution with a similar proficiency to what was seen pretraumatically.

Comparing the task performance of such recovered individuals to non-injured controls one may draw the faulty conclusion that the task solutions in question are not only of similar proficiency but also identical. Only in detailed experimental analysis (e.g., Mogensen and Malá, 2009; Mogensen, 2011b), it will become clear that apparently similar surface phenomena are achievable via significantly different neural and cognitive mechanisms – “strategies.”

## The REFCON Model

The REF model primarily addresses the neurocognitive mechanisms associated with problem solving. The overall principles described by the model are, however, believed to be of a more general nature and are likely to be involved in neural and cognitive mechanisms associated with other domains than those of problem solving (e.g., Mogensen, 2012b). Partly on the basis of such assumptions, we have (Overgaard and Mogensen, 2014, 2015) developed a new variant of the REF model called REFCON. The REFCON model addresses mechanisms associated with perceptual analysis and conscious awareness. And offers a framework presenting aspects of the NCC within a broader model of perceptual processes.

Various cognitive domains have, traditionally, after brain injury mainly been evaluated as being impaired or unaffected. Such a dichotomy may, however, be too simple to represent the actual situation. For instance, brain injured patients often demonstrate a reduced rather than absent manifestation of the impaired cognitive domain. Working memory is reduced but not absent (McAllister et al., 2006) and patients with prosopagnosia do not experience “nothing” where a face should have been but rather “something unfamiliar” (Oakley and Halligan, 2013). It has also been established that even in intact individuals the performance on neuropsychological tests represent a spectrum rather than a fixed “normal” level of performance (e.g., Schretlen et al., 2003; Binder et al., 2009). Consequently, under normal as well as pathological conditions the level of cognitive proficiency is best evaluated as a given position within a broad spectrum. As mentioned above, an absolute dichotomy between conscious and non-conscious states also seems unlikely on the basis of studies applying more detailed measurement techniques in various experiments on consciousness (for reviews see, e.g., Overgaard and Mogensen, 2014, 2015; Sandberg and Overgaard, 2015). In this respect there is a clear parallel to what is found within many and maybe all aspects of cognition. In general, we see many parallels between what is addressed by the REF model within problem solving and what is found regarding consciousness.

To illustrate some of these parallels, we will present examples of results found in the study of blindsight. By “blindsight,” one normally understands a “visual capacity in the absence of acknowledged awareness” after lesions of the primary visual cortex (Weiskrantz, 1986). Like in cortical blindness or hemianopia, there is apparently no subjective awareness of visually presented stimuli within the affected field. In spite of this, certain visual functions appear to be preserved. In some cases, what is preserved may be the ability to indicate the position of a visually presented object within the affected field. In other cases one or more of the other aspects of the visually presented object appear to be available to behavioral control. The crucial aspect in case of blindsight seems to be the very segregation of functional and phenomenal aspects of mental states (e.g., Riddoch, 1917; Pöppel et al., 1973; Weiskrantz et al., 1974).

In any study of blindsight, two aspects are of crucial importance: (A) whether or not conscious awareness is present, and (B) the test of behavioral/cognitive demonstration that at least some aspect(s) of the visually presented information within the affected field is/are able to influence behavioral control.

According to a strict definition, blindsight is only demonstrated if A provides evidence of no conscious awareness while B reflects the ability of visually presented information to influence behavior (Overgaard, 2011, 2012). Addressing these issues, we see a number of parallels to some of the studies mentioned above in the context of problem solving by brain injured individuals. In case of the determination whether or not conscious awareness is present, the subject is traditionally presented with a “yes-or-no” option – having to report either being conscious of the stimulus or not being conscious of what is visually presented. But in a parallel to the need of addressing “how” rather than just “whether-or-not” an individual is posttraumatically able to solve a particular task, there may also be a need for a more thorough and graded evaluation of conscious awareness.

A detailed analysis of the level of conscious awareness can be obtained using the PAS developed by Ramsøy and Overgaard (2004). Rather than the dichotomous reports used in most blindsight studies, PAS provides an evaluation of the degree of conscious awareness of an individual in a given situation. Such situations may, for instance, be studies of subliminal perception in normal individuals (Overgaard et al., 2006, 2013). And using PAS rather than dichotomous reports, it appears that there is a strong correlation between the behavioral performance and the reported clarity of experience in various experimental setups (Koch and Preuschoff, 2007; Sandberg et al., 2011). When using PAS in a case of blindsight it was found that the patient GR demonstrated a pattern of test results differing from what is expected in traditionally defined blindsight (Overgaard et al., 2008). Rather than behavioral utilization of visual stimuli unavailable to conscious awareness, GR showed no behavioral utilization of stimuli which according to PAS were completely unavailable to conscious awareness. In contrast, GR had various degrees of behavioral reactions to stimuli which were – according to PAS – consciously available to various degrees. That is, utilizing this more detailed analysis, blindsight was not present in the strict definition of stimuli being able to direct behavior in the complete absence of conscious awareness.

We believe that results obtained in studies of blindsight patients can be understood in terms of and explained by the mechanisms described by the REF model. This has been more thoroughly discussed elsewhere (e.g., Overgaard and Mogensen, 2014) but a few examples will be mentioned briefly.

Studies of blindsight have demonstrated that rather than being a weakened version of the normal visual experience, the visual “perception” occurring in blindsight may at least in some instances be a reflection of information processing strategies different from those seen normally. In a signal detection analysis of the yes/no detection judgements and forced choice detection tasks of the patient GY it was found that his sensitivity was significantly higher in the forced choice task (Azzopardi and Cowey, 1997). The same patient was able to match some but not all aspects of visual stimuli when comparing stimuli presented in the blind and healthy hemifields (Morland et al., 1999). Color and motion were associated correctly, but – in contrast – brightness was not. In both of these instances the indication appears to be that rather than being a weakened version of the normal processing strategies, the demonstrated blindsight reflects a different type of “strategy.”

Based on findings in blindsight, other types of normal as well as pathological perceptual phenomena and various studies addressing conscious awareness, we have proposed the REFCON model as a framework for understanding perceptual processes in general and the mechanisms of conscious perceptual awareness in particular (Overgaard and Mogensen, 2014, 2015).

The REFCON model is based on the same types of EFs, AMs, and ASs as the original REF model. And crucial mechanisms within the REFCON model are the reorganizations and backpropagation mechanisms described in the REF model. There are, however, a number of more specific elements to the REFCON model. An important element within the model is the perceptual elementary function (PEF) which differs from other EFs by receiving a more or less direct sensory input. In general, EFs do not have any functional specificity reaching beyond their basic information processing. An EF is associated with a particular cognitive domain only to the extent that it is integrated in an AS associated with that domain. In other words, the cognitive domain is exclusively determined by the changing input/output relationships of the EFs. In contrast, a PEF is “prewired” to be associated with a sensory modality and thus with perceptual analysis.

The PEFs are likely to be located at subcortical as well as cortical levels. In case of vision a primary cortical localization is within the V1/V2 region (although mediation of visual PEFs is not the only role of this cortical area). The PEFs are central processing steps for the incoming sensory information.

Sensory information within a given modality activates a pattern of PEFs associated with that modality. The next step, however, reaches beyond the PEFs and includes activation of perceptual algorithmic modules (PAMs). PAMs are AMs, like those described above. Patterns of interconnected EFs (in case of PAMs including PEFs) and established via experience/backpropagation mechanisms. Like other AMs, the PAMs are not themselves the basis for neither behavioral manifestations nor conscious awareness. The PAMs are hierarchically arranged and normally a perceptual analysis progresses through a substantial number of layers of PAMs.

When a pattern of PEFs is activated, this pattern will lead to a degree of activation of a number of PAMs of the lowest level of the hierarchy. When thus activated, each of these PAMs “interrogates” each of its constituent PEFs as to its level of activation. Out of the activated PAMs the one which in such an interrogation reaches the highest level of correspondence between constituent and activated PEFs, respectively, is the one to reach a full level of activation. In the hierarchy of PAMs, the PAMs of the lowest level are constituent elements within higher levels. As the perceptual process progresses through the levels of PAMs, the selection process is similar to the one determining which PAM of the lowest level becomes fully activated. Only the PAM of the highest level of the above-described hierarchy is (potentially) available to the regulation of behavior and/or conscious experience.

**FIGURE 2:**
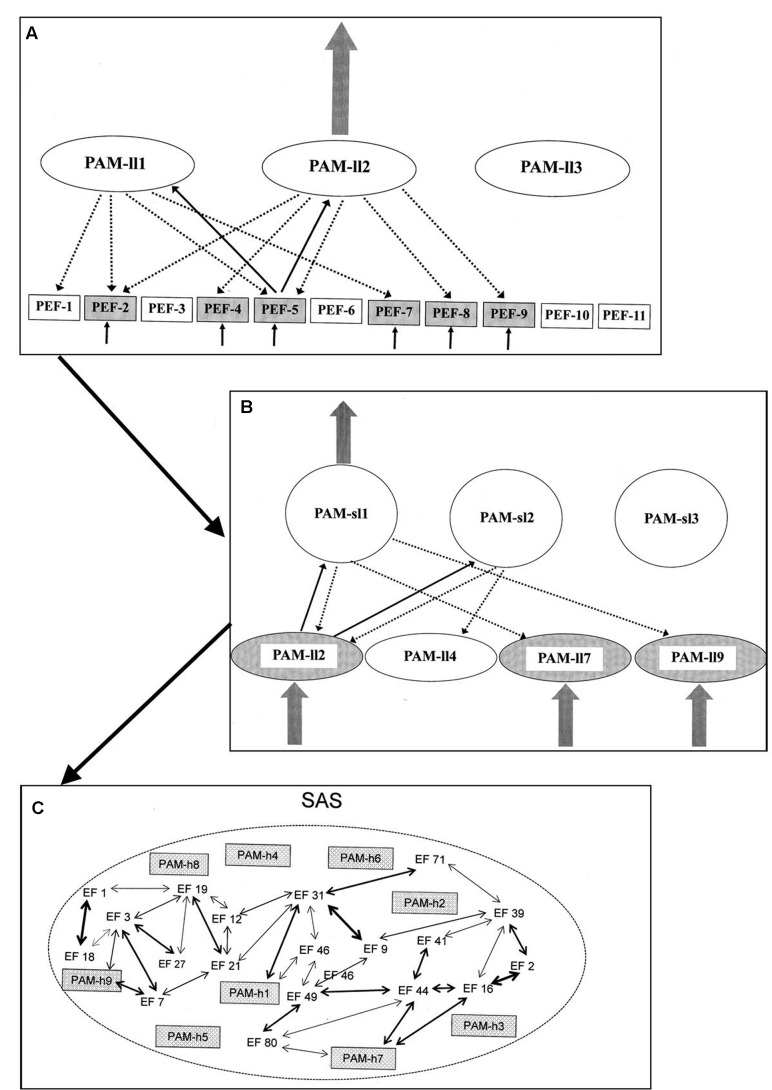
**Simplified illustration of the process (according to the REFCON model) from activation of PEFs to integration in SAS [see the present text and Overgaard and Mogensen (2014) for details – including explanations of A–C]**.

The PAMs of the highest level will – when fully activated – potentially become integrated into a unique form of AS named the situational algorithmic strategy (SAS). The SAS is a highly dynamic network reflecting the current status of the individual. The SAS combines elements within sensory/perceptual and a broad spectrum of other dimensions relevant to the current status of the individual. Only when integrated into the network of SAS will the PAMs of the highest level be available for (1) cognitive access, (2) behavioral control, and (3) conscious awareness. The degree and pattern of the integration into SAS determines the level of availability for all three elements. A simplified illustration of the process from activation of PEFs to integration in SAS is given in **Figure 2**.

Within the framework of the REFCON model, the injury to the V1/V2 area in blindsight is seen as injury to the neural substrates of PEFs crucial to vision. In such a situation, however, preserved PEFs (cortical and/or subcortical) may still lead to activation of PAMs. The activating patterns and modules are, however, different from those which could have occurred without the injury.

The rather rudimentarily analyzed visual information available in blindsight is in this context seen as a reflection of a process in which the visual analysis has progressed through a relatively low number of levels of PAMs. Consequently, the analysis will reach its most advanced level – the highest level – at a comparatively earlier stage of analysis than what is normally seen. In order to become integrated into SAS – and thereby made available to behavioral control and potentially a level of conscious awareness – such a process requires an unusual degree of top-down control and effort. Blindsight is therefore – according to the REFCON model – a manifestation of a process in which SAS is “top-down” modified in such a way that PAMs of a relatively low level will nevertheless become integrated into SAS. The nature of such a process will be further addressed below.

Blindsight patients may over time (and to an extent also in a situationally dependent manner) exhibit different levels of conscious awareness of the visually presented stimuli. According to the REFCON model this can be explained as dynamically changing degrees of integration of PAMs into SAS. Different situational demands can cause such modifications in network integration. Experimental setups may differ in their implicit or explicit demands on making visual information within the affected field available to behavioral control and/or a level of conscious awareness. The effects will be different levels of top-down influences on the integration of PAMs into SAS. Furthermore, it has been demonstrated that training and learning processes also need to be taken into consideration. Both in animal models (Humphrey, 1974; Dineen and Keating, 1981) and in blindsight patients (Zihl, 1980; Bridgeman and Staggs, 1982; Zihl and Werth, 1984; Stoerig, 2006; Henriksson et al., 2007; Raninen et al., 2007; Chokron et al., 2008) training can increase the degree to which stimuli are available to behavioral control and to subjective awareness (Sahraie et al., 2006). Such demonstrations of learning effects agree well with the REF associated reorganizations of relevant networks of EFs and PEFs – be it within PAMs or elsewhere.

Since the integration of PAMs into SAS is the crucial process regarding making the information available to consciousness, the REFCON model points to a NCC associated with such a task-dependent and distributed process. The model also emphasizes the need to see the NCC as an aspect of more general neurocognitive processes (as stressed in the “Introduction” of the present paper).

Although consciousness and cognition may be rather clearly conceptually differentiated, it remains undetermined whether or not cognitive processes occur in the complete absence of consciousness (as argued by, e.g., Hassin, 2013). As mentioned, instances of for example “blindsight” in the apparent absence of conscious awareness may not reflect cognition without consciousness – but rather the presence of consciousness at such a low level that if simplified into a simple “yes/no” response it would be reported as no consciousness at all. According to the REFCON model it is the integration into SAS that makes a PAM available to behavioral/cognitive control as well as to conscious awareness.

It may be relevant to stress that – although part of a perceptual process – the PAMs of the higher levels typically represent a broad range of information regarding what is being perceived. Clearly the structure of the PAMs allow the strictly perceptual analyses – e.g., identifying something in sight as being a dog, a face or a piece of furniture. But all PAMs are structured by experience. And via experience additional “purely semantic” information may also be represented within a PAM of the higher levels. Within the activated PAM additional “meaning” (semantic knowledge) may be (via integration into SAS) brought into conscious awareness. The perception of the face can, for instance, be accompanied by the immediate awareness of the identity of and prehistory with the individual in question – without the need for separate recruitment of semantic and/or episodic information.

## Shortcomings of Domain-Specific Computational Models

Practically all neurocognitive models including a computational level focus on a more or less restricted neural and/or cognitive domain. The REF and REFCON models attempt to describe and explain the “input and output” related systems as well as processes more distal to the sensory input or motor output of the brain. The models do, however, focus on somewhat limited cognitive domains: the REF model primarily on problem solving and the REFCON model on perception/conscious awareness.

Since even models with such a “limited” neural/cognitive scope tend to be extensive and complicated, it is understandable that such limitations are imposed. A shortcoming of practically all such models is, however, that none of the cognitive domains (nor their neural substrates) functions in isolation. In order to understand the mechanisms of processes within any such domain, one needs to include inputs/influences from systems and mechanisms partly found outside the scope of a given model.

One such example can be found in the above description of the REFCON model. When discussing how the rather rudimentarily processed information within the affected field in blindsight may still reach a level at which it can influence behavior and potentially even be (at least somewhat) available to conscious awareness, we stress the influences of top-down processes. Top-down influences in the form of for instance experimental instructions may cause PAMs which remain at a relatively “low” level of the normal hierarchy of PAMs to become integrated into SAS – thereby influencing behavior and potentially conscious awareness. But such an explanation begs the question: In which way do these top-down influences exert their influence? And for that matter: How is a top-down influence to be conceptualized in the context of the REF mechanisms?

**FIGURE 3:**
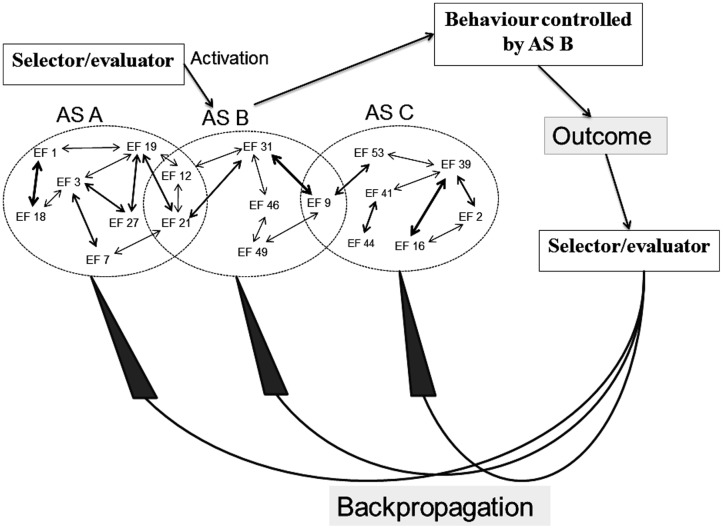
**Simplified illustration of the mechanisms (according to the REF model) of selection and evaluation (with consequent backpropagation-mediated modifications) of algorithmic strategies [see the present text and, e.g., Mogensen (2014) for further explanations]**.

In a similar manner, the original REF model contains elements which remain underdefined. As illustrated in **Figure 3**, a selector/evaluator mechanism has important roles within the mechanisms of activation as well as reorganization of the ASs. When an individual faces a “problem situation” (defined as any situation in which a task has to be solved), the selector mechanism will activate an AS which then becomes the mechanism of the behavior in that situation. If the situation has previously been associated with a (still existing) AS, then that AS is the one activated and presumably leading to a successful task solution. If, however, the situation is “novel” – be it so far never encountered or a situation previously associated with an AS which has now been lost to injury or disease – the selector mechanism will sequentially activate ASs associated with more or less similar situations.

Whenever an activated AS causes a behavioral manifestation, the outcome of that manifestation is evaluated by the evaluator mechanism. The outcome of that evaluation leads to the backpropagation mechanism modifying the connectivity between EFs. This reorganization of the interplay between EFs causes modification of existing ASs as well as (potentially) the creation of novel ASs.

It has been stressed that the selector/evaluator mechanism should not be conceptualized as a unitary entity (e.g., Mogensen, 2012b, 2014). And that the selector/evaluator mechanism should (like ASs) be considered a network of interconnected EFs. Nevertheless, the nature of the selector/evaluator mechanisms and its relationship to processes such as motivation and perception has remained relatively ignored in the accounts of the REF model.

Only when considered within an even broader account of neurocognitive organization can models such as the REF and REFCON gain a better conceptualization of processes such as those mentioned above. And the NCC be understood in a broader context. Only a computational and integrative model of the entire neurocognitive organization of the brain will ultimately be able to solve all such problems.

Additionally, only a more comprehensive neurocognitive model is able to serve as a framework for experimental design and data interpretation in the context of connectome research.

Presently, we propose the REFGEN model as a framework within which such a broader computational approach to neurocognitive organization can be achieved. The REFGEN model also allows consciousness (and the NCC) to be modeled as an integral part of all dimensions of cognition and its neural substrate. The REFGEN model is a further elaboration of the REF and REFCON models rather than a completely new construction. The REFGEN model operates with the same fundamental principles and elements as the REF and REFCON models.

## The REFGEN Model

The neural mechanisms mediating homeostatic control within the basic and vital regulatory systems of, e.g., body temperature and food intake/energy balance share (although with major variations) a common principle of structure (e.g., Nakamura, 2011). A crucial element is a set-point relative to which the current status is evaluated. Depending on the system in question, the set-point may be highly stable or relatively influenced by external and internal factors. Another crucial element is the detection systems and representations of the current status within the domain in question. Finally, mechanisms able to move the current status in the direction of the set-point are available. Anatomically separate mechanisms are at least in some instances involved in mediation of purely physiological responses (e.g., Satinoff and Rutstein, 1970; Van Zoeren and Stricker, 1977) and behavioral regulatory mechanisms (Satinoff and Shan, 1971; Van Zoeren and Stricker, 1977), respectively.

Although such regulatory systems are of a basic and relatively simple nature, we believe that they share important features with the regulatory mechanisms associated with such “higher” functions as problem solving, perception, conscious representation, and the mediation of mental phenomena in general. Consequently, the framework proposed in the REFGEN model also contains the four crucial elements of:

(A) A representation of the “goal” (“set-point”) or “expectation” toward which it is desired to progress.
(B) A representation of the current status.
(C) A comparison mechanism detecting discrepancies between the status of what is described under “A” and “B.”
(D) Mechanisms which can be activated to move the current situation in the direction of what is indicated under A.

While the processes described in “A–D” have a lot in common with the basic processes mediating simple homeostasis, the mechanisms of the REFGEN model are of an extremely dynamic nature and reorganizations via backpropagation mechanisms are of crucial importance.

**FIGURE 4:**
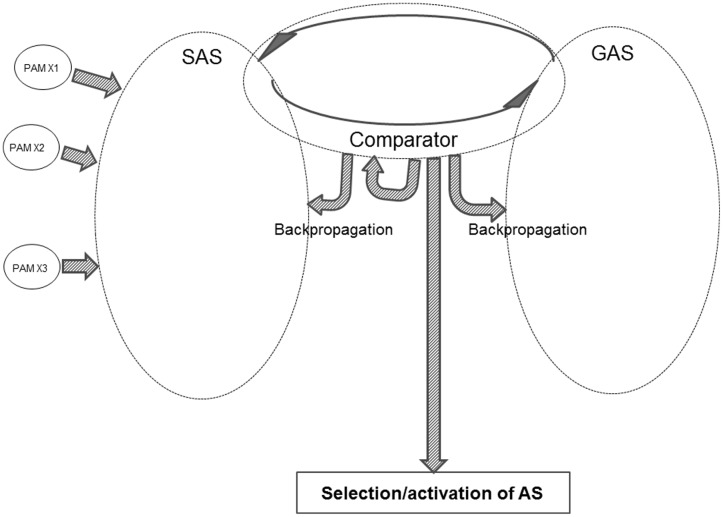
**The major components of the REFGEN model (see text for details)**.

**Figure 4** offers a sketch of the major components within the REFGEN model.

One of the three major elements within the model is SAS – the situational algorithmic strategy – which was already introduced in the REFCON model (see above). SAS is the equivalent of the above-mentioned “B” component: a representation of the current status. SAS reflects the current status of the individual, including the available information about the current proximal and distal environment – as well as the internal status of the individual.

Within the REFGEN model, the representation of the “A” component (the “goal” or “set-point” toward which it is desired to progress) is the goal algorithmic strategy (GAS). Like SAS, GAS is a highly dynamic and widely distributed AS. GAS consists of a constantly changing pattern of interconnections between EFs. While the connectivity within SAS changes to reflect the current status (external as well as internal) of the individual, GAS in a similar manner changes to reflect the goal toward which it is desired that the individual currently moves. Such goals can be immediate and short-lived. An example could be to immediately collect information within a particular part of the visual field. Or the goals can be of a more general and distal nature – such as taking steps toward an educational, professional, or personal goal.

The third major component within the REFGEN model is the Comparator. The Comparator mechanism constantly performs a two-way comparison between the status/structure of SAS and GAS, respectively. Doing this, the Comparator is the parallel to the above-described “C” component: a mechanism constantly comparing the current situation/status (SAS) to the current “set-point” or goal (GAS).

As indicated in **Figure 4**, the activity of the Comparator can lead to selection and activation of ASs. Thereby behavioral/mental surface phenomena are elicited. The Comparator can also change the structure within SAS, GAS and/or the Comparator itself via backpropagation mechanisms. These selections/activations of ASs as well as the backpropagation mediated changes of SAS are the counterpart to the “D” component: the mechanisms that may move the current situation in the direction of the “set-point” or goal.

**FIGURE 5:**
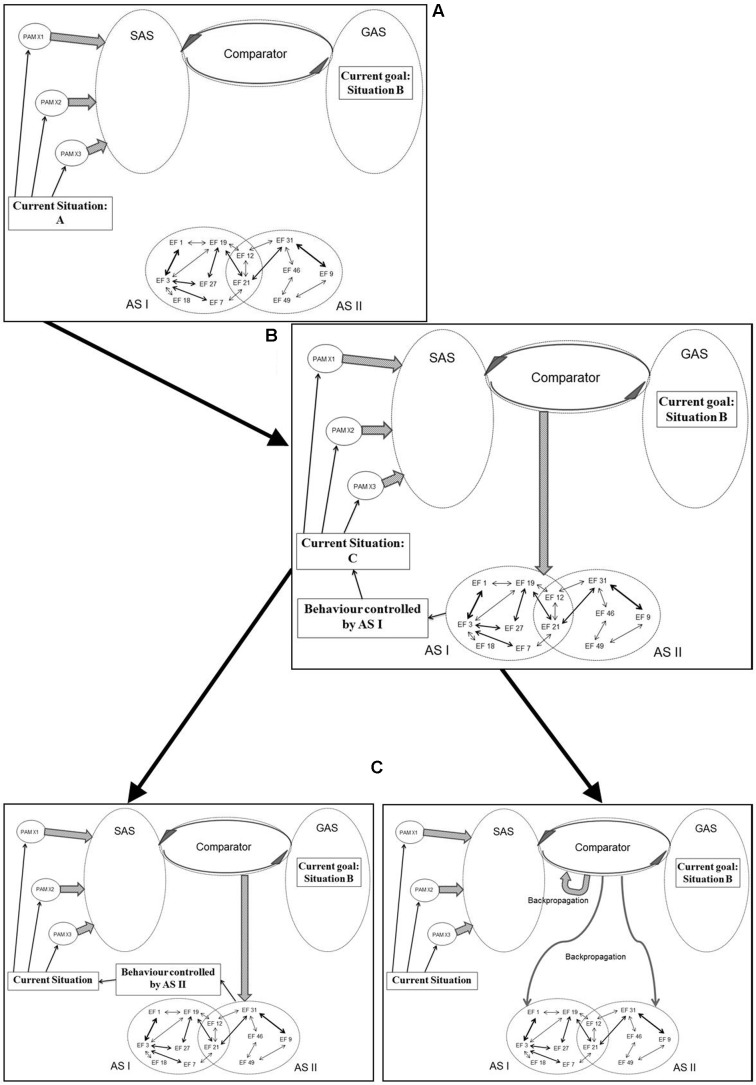
**Illustration of how processes originally described in the REF model (and illustrated in **Figure 3**) are realized within the REFGEN model (see text for details – including explanations of A–C)**.

**Figure 5** illustrates an example of how processes originally described in the REF model (and illustrated in **Figure 3**) are realized within the mechanisms of REFGEN. The individual is faced with a problem solving situation. The current situation is “A” while the desired goal (the solution to the problem) is situation B. In a spatial orientation task, “A” might be the current position of the individual while “B” is the goal position to be reached.

Being in situation A the individual receives sensory information characterizing the situation and according to mechanisms described by the REFCON model a representation of the current situation will become part of SAS. Simultaneously, the current goal of reaching situation B constitutes part of GAS. A number of potentially relevant ASs (simplified as AS I and AS II) are available. The discrepancy between the current situation (A) represented within SAS and the goal situation (B) represented within GAS is detected by the Comparator. Consequently, the Comparator activates mechanisms potentially able to change the current situation into something closer to “B” (ideally “B”). As illustrated in panel B of **Figure 5**, the Comparator activates AS I as such a potential mechanism. Activation of AS I leads to the behavior mediated by this AS and in the present example this leads to a change of the current situation from situation A to situation C. Via the REFCON mechanisms, situation C now becomes represented within SAS as the current situation. Once again, the Comparator registers a discrepancy between the current situation (C) and the goal situation (B). This leads the Comparator to activate another AS (AS II) and to the behavior mediated by AS II. Consequently, a new current situation is achieved and subsequently represented within SAS. But as illustrated in the right panel C of **Figure 5**, the Comparator, in parallel, activates a number of backpropagation processes. These backpropagation processes modifies activated as well as non-activated ASs (in the current example AS I and AS II) and also modifies mechanisms within the Comparator itself. The latter modifications are primarily associated with the degree to which a particular problem situation is associated with activation of a particular AS. If, for instance, AS I achieved a partial solution to the present problem, activation of AS I in similar situations will continue to be attempted. In contrast, if situation C is even further from the goal situation B than the original situation A, future activation of AS I in similar situations will become less likely.

**FIGURE 6:**
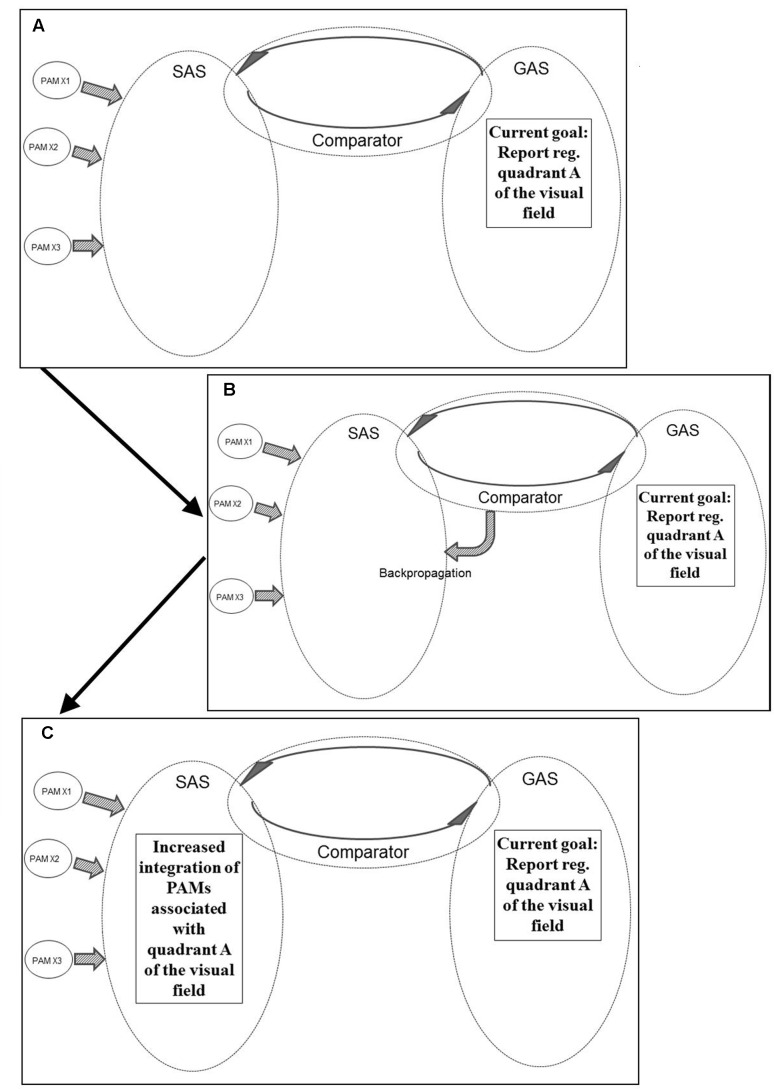
**Illustration of REFGEN mechanisms associated with a situation in which an individual is to report stimuli within a given quadrant of the visual field (see text for details – including explanations of A–C)**.

**Figure 6** illustrates another example of REFGEN mechanisms. Within GAS there is a representation of a current goal which consists in a demand to report sensory information within quadrant A of the visual field. Consequently, the Comparator (as illustrated in panel B of **Figure 6**) activates backpropagation mechanisms modifying the structure of SAS. These modifications (as illustrated in panel C of **Figure 6**) increases the likelihood of integration of PAMs associated with visual information originating within quadrant A of the visual field.

Prior to the Comparator initiated backpropagation modifications of SAS, visual information within quadrant A did have the potential of becoming sufficiently integrated into SAS to reach a level of conscious awareness and consequently become reported. With the backpropagation mechanism illustrated in panel B of **Figure 6**, however, the structure of SAS becomes more susceptible to PAMs associated with the relevant part of the visual field. Consequently even less highly processed PAMs may become sufficiently integrated into SAS to reach conscious awareness and/or behavioral control. As discussed above, this is the situation in which top-down influences may cause even relatively low level processed PAMs to become available for conscious awareness and/or behavioral control.

**FIGURE 7:**
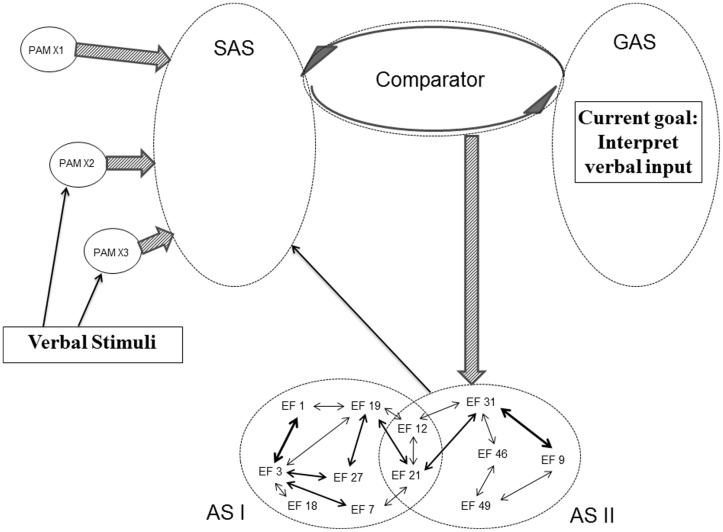
**Illustration of REFGEN mechanisms associated with interpretation of verbal input (see text for details)**.

The structure of SAS does not only have the potential of being modified by backpropagation mechanisms initiated by the Comparator and by the PAM integration described in the REFCON model. Activated ASs are also able directly to modify the current structure of SAS. Already the initial REF model emphasizes that the surface phenomena occurring as a consequence of AS activation can either be behavioral manifestations or mental/conscious representations (Mogensen and Malá, 2009; Mogensen, 2011a,c). And in the framework of REFGEN, the mechanism of such mental representations is an AS mediated modification of the structure of SAS. **Figure 7** illustrates an example of such a process. In that situation there is, within GAS, a representation of a current goal of interpreting verbal input. And some of the PAMs becoming more or less integrated within SAS represent linguistic elements. In such a situation, the Comparator will respond to the discrepancy between uninterpreted linguistic elements (represented within SAS) and the goal of interpreting verbal input (represented within GAS) by activation of ASs with the potential of leading to an interpretation/conscious representation of the received verbal message. In the example offered in **Figure 7**, AS II is an AS which as its mechanism has a direct modification of the structure within SAS – potentially leading to the desired linguistic interpretation. In case activation of AS II does not cause a satisfactory interpretation (as evaluated by comparison between the status of SAS and GAS), the Comparator will subsequently activate other ASs – along the lines illustrated in **Figure 5** (but with the modification that outputs of the ASs in questions will not be overt behavior but restructuring of SAS).

**FIGURE 8:**
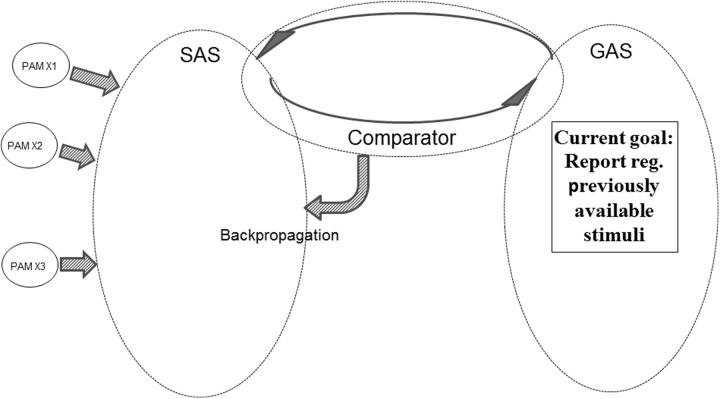
**Illustration of REFGEN mechanisms associated with a situation in which an individual needs to report about previously available stimuli (see text for details)**.

**Figure 8** illustrates another aspect of the REFGEN mechanisms. Within GAS there is a representation indicating the need to report (or at least mentally represent) previously available stimuli – e.g., a picture seen 2 h ago. Since a representation of the picture in question is not presently available in SAS, the Comparator will activate backpropagation mechanisms changing the structure of SAS in the desired direction. The direction in question is integration of previously activated PAMs representing the previously presented picture. Until the desired pattern of PAM activation has been achieved, the Comparator will continue backpropagation mediated modifications of SAS. In such a situation GAS does, of course, not contain a representation of the requested information (e.g., the previously seen picture). Rather, the constellation of interconnected EFs within GAS represents a demand to reconfigure SAS in such a way that a given task can be solved. That might be to select the correct door out of three or to provide a verbal report regarding what was seen in a given situation. In both cases solving the task may require integration of PAMs representing the picture into SAS. And such a situation would only be achieved by Comparator mediated changes of the configuration of SAS – Comparator working to achieve a correspondence between SAS and the “demands” represented in GAS.

**FIGURE 9:**
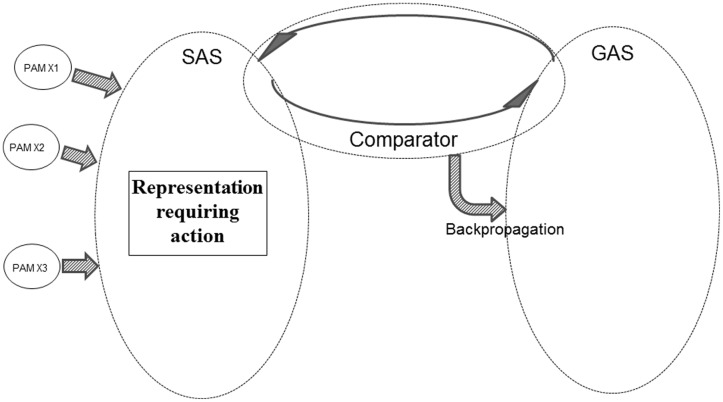
**Illustration of REFGEN mechanisms associated with a situation in which a novel representation within SAS requires an action not presently represented as a goal within GAS (see text for details)**.

If SAS contains a representation requiring one or another kind of action (e.g., a shift in perceptual focus, a verbal demand requiring overt behavior or the need to recall specific information) the Comparator will perform a comparison against the current goals represented within GAS. If, as illustrated in **Figure 9**, GAS does not contain a representation of such a goal, the Comparator will initiate backpropagation mechanisms modifying GAS, until the required action is represented within GAS.

The above examples of mechanisms within the REFGEN model are only meant to highlight some of the aspects of this framework. It should be emphasized that none of these mechanisms is to be seen in isolation. Rather, they are closely integrated and interacting serially as well as in parallel. Within the examples given above, for instance, a number of the described mechanisms can easily be combined into a simple serial sequence. An experimental subject is placed in a test situation with the expectancy of receiving verbal instructions. When these instructions are delivered, the mechanisms illustrated in **Figure 7** take effect. The subject is expecting (= having a representation of this goal within GAS) verbal instructions that must be interpreted. Upon interpretation of the instructions, the SAS of the subject contains a representation of a demand to report about stimuli delivered within a particular part of the visual field. Since the GAS of the subject does not already contain a representation of such a goal/demand, the mechanisms illustrated in **Figure 9** take effect and the Comparator accomplishes a modification of the structure of GAS – leading to a representation within GAS that the subject is to report about stimuli within a particular part of the visual field. This leads to the situation illustrated in **Figure 6** and the mechanisms within that example take effect – leading to a Comparator mediated modification of SAS in the direction of increased integration of PAMs associated with stimuli within the particular part of the visual field. This combination of examples could in principle be expanded endlessly – for instance to include a situation in which the subject is later confronted with a new phase of the experiment. In this new situation the subject is to report about the previously presented stimuli. That would lead to activation of the mechanisms illustrated in **Figure 8**: the Comparator mediated modification of SAS in order to (as far as possible) re-represent the previously presented stimuli and report about these.

For the sake of simplicity the above given combination of examples has restricted itself to serial combinations. In real-life situations, however, numerous factors will constantly modify the structure of SAS as well as GAS – and the Comparator will perform numerous simultaneous comparisons as well as modifications. Thus, numerous factors will influence mechanisms which if viewed in isolation may appear relatively simple.

Although our description of the REFGEN model deliberately avoids most of the traditional psychological terminology in terms of references to specific cognitive domains, the processes described by the model are obviously related to numerous such domains. For instance, the sequence of events illustrated in **Figure 6** includes what in traditional terms would be called attentional processes. A task requires a subject to report about visual stimuli from a specific part of the visual field and the neurocognitive systems of that subject ensures an increased integration of visual information regarding the specific part of the visual field. In more traditional terms this could be referred to as a process of directing visual attention to a specific part of the visual field. The processes illustrated in **Figure 8** aim at achieving reports about previously available stimuli. In traditional terms, a task requiring recall of information previously stored in long-term memory.

## The Neural Substrate of the REFGEN Mechanisms

As previously described, all ASs are dynamically changing structures. Some undergo frequent modifications while others – once constructed in an adequate constellation – receive comparably less modifications (e.g., Mogensen, 2011a,c). While the structure remains unchanged within the individual EFs, the longer-range connections combining EFs into an AS are the sites of these dynamic reorganizations.

The SAS, GAS, and Comparator of the REFGEN model are highly specialized ASs. All three are widely distributed in the brain. And all three are constantly undergoing a high level of modifications. They are modified in ways described above – and partly by the REFCON model (Overgaard and Mogensen, 2014, 2015). SAS and GAS are likely to be the ASs faced with the highest demand regarding rapid and constant reorganizations. Thus, the necessary plastic mechanisms have to involve rapid and quickly reversible changes. A candidate for at least one of these mechanisms is the dynamic modifications of dendritic spines (e.g., Trachtenberg et al., 2002; Pilpel and Segal, 2004; Holtmaat et al., 2005; Majewska et al., 2006; Dent et al., 2011; Yuste, 2011; Bosch and Hayashi, 2012; Araya et al., 2014) and potentially the activation/deactivation of “latent” synapses (e.g., Kaas, 1991; Kerchner and Nicoll, 2008) – synapses with the ability of rapid oscillations between “on” and “off” via for instance local GABAergic mechanisms (e.g., Jones, 1993; Chen et al., 2002).

SAS and GAS are both highly distributed and elements of both SAS and GAS are likely to be found within almost all parts of the brain (cortically as well as subcortically). Furthermore, relative to each other, SAS and GAS are likely to have a “parallel” distribution – in the sense that within most of the neuroanatomically defined brain structures the neural substrate of EFs will have at least partly overlapping elements associated with SAS and GAS, respectively. It should be remembered that in the “homeostatic” process of bringing a correspondence between the current condition of SAS and GAS (see above) the “ultimate goal condition” can be described as a complete identity between the structure of SAS and GAS, respectively.

The details of the anatomy and physiology of SAS and GAS remain to be explored in future research. But as a point of departure we imagine a connectivity in which practically all EFs are “pre-connected” to be part of both SAS and GAS. This pre-wiring is, however, via potentially the mentioned “latent” synapses. That is: within each EF one will (besides the internal circuits mediating the information processing provided by that EF) find separate relatively long-range connections with the ability to quickly integrate the EF in question into SAS and GAS, respectively (both types of connections being present in all EFs) – or to disconnect the EF from SAS or GAS. It is also via the on/off mechanisms of such synapses that the integration of the PAMs of the highest level into SAS (see above) may take place.

Schematic presentations such as **Figures 2**, **4**, and **7** may leave the impression that SAS, GAS and for instance PAMs and various other ASs are anatomically separate. The separation is, however, more conceptual than anatomical. All ASs (including SAS and GAS – as well as Comparator) and AMs (including PAMs) are composed of EFs (as well as the interconnections between EFs) and all EFs can in principle become part of all ASs and all AMs. Consequently, an anatomically more correct illustration of the neural substrate of SAS, GAS, Comparator, and other ASs and AMs would be an immensely large matrix within which for instance SAS and GAS were represented by constantly changing and significantly overlapping patterns.

This anatomical overlap also points to possible physiological mechanisms for some of the processes described above. When, for instance, PAMs of progressively higher levels become more and more integrated into SAS (see above) this is a process dominated by the engagement of a progressively more elaborate network of EFs being “linked” into SAS via activation of the mentioned synaptic “potential SAS connections” – synaptic connections already present (in “latent” or active form) within the neural substrate of the individual EF. Of course many of the EFs participating in such a PAM are likely already to be participating in SAS due to being part of for instance other AMs. It is then the coordinated activation within a given PAM that solves the “internal binding problem” within SAS.

Within the neural substrate of an EF there are also specific mechanisms providing output from the EF regarding its status relative to both SAS and GAS. These mechanisms provide input into the dedicated Comparator network of EFs. These “status” signals from a given EF to Comparator may take the forms:

(1) Neither part of SAS nor GAS.
(2) Part of SAS but not GAS.
(3) Part of GAS but not SAS.
(4) Part of both SAS and GAS.

Since Comparator as mentioned above works toward a (hypothetical) situation characterized by identical patterns within SAS and GAS, signals reporting condition 2 and 3 may be seen as “error signals” – calling for one or another type of action (be it behavioral activities or restructuring of SAS, GAS, or both).

The (constantly changing) EF-constellation and consequently information processing within Comparator mediates algorithms having a general form of:

IF (A [constellation of error signals] AND B [configuration within SAS] AND C [configuration within GAS]) THEN D [action].

The action (D) “initiated” by Comparator is activation of an AS and/or backpropagation mediated modifications of SAS, GAS, and/or Comparator. If an AS is activated the identity of that AS is determined by the parameters A–C.

In order to be in a position to achieve such actions, the neural substrate of Comparator needs to be equipped with efferent connections reaching the neural substrates of the EFs constituting SAS, GAS, and other ASs. Comparator is a dynamically changing system of interacting EFs and practically any EF may become part of Comparator. Consequently it should be expected that the neural substrate of all EFs has efferent projections (direct or indirect) to the neural substrate of all other EFs.

If parameter A (the constellation of error signals) constitutes a significant either reduction or increase on a relatively short time scale, parameter D (action) is likely to include an activation of neural (including dopaminergic) activations associated with success or failure, respectively (e.g., Nakahara et al., 2004; Bayer and Glimcher, 2005) – signals likely to interact with the backpropagation processes (e.g., Schiess et al., 2016).

It has repeatedly been argued that the traditionally applied backpropagation algorithms (e.g., Werbos, 1974, 1994; Rumelhart et al., 1986) may not be biologically plausible (e.g., Stork, 1989; Mazzoni et al., 1991a,b). It has, however, also been stressed that improved insights into the physiology of biological networks may prove such learning rules to be biologically plausible (e.g., Stork, 1989). Crick (1989) has stressed the need for a “brain-like” algorithm that produces the same outcome as backpropagation and O’Reilly (1998) has outlined a series of areas within which computational models of cortical information processing should be evaluated. Several further developments of the original backpropagation algorithm have been evaluated to be more biologically feasible (e.g., Durbin and Rumelhart, 1989; O’Reilly, 1996). Not the least the “GeneRec” model of Hinton and McClelland (1988) appears to have substantial biological merits (e.g., O’Reilly, 1996, 1998). Only future mathematical modeling as well as biologically oriented scrutiny of the REFGEN model will allow us to identify which backpropagation mechanisms are the most plausible in the present context. Many of the relevant processes have to do with comparisons between and adjustments regarding discrepancies between SAS and GAS. And many of these SAS/GAS processes take place rather “locally” – within a variety of rather restricted structures (e.g., regions of the neocortex) where SAS and GAS as mentioned are found in a more or less “parallel” organization. In this context it may be relevant to consider the results of Schiess et al. (2016) who demonstrated that a backpropagation algorithm may be successfully implemented within a single neuron equipped with non-linear dendritic processing (e.g., a neuron crossing the layers within a region of the neocortex). In their analysis Schiess et al. (2016) also demonstrated that a reward signal may modulate these backpropagation mechanisms.

Clearly all aspects of the REF and REFCON models are integrated into and to an extent reconceptualized within the REFGEN model. To provide but one example: when “blindsight” is obtained via the already mentioned top-down modifications of SAS, this process is an interaction between GAS and SAS. The instruction to the patient (to “guess” regarding a stimulus within the “blind” part of the field of vision) leads to a representation of that task within GAS (of course via processes involving verbal interpretations and modifications of SAS – see for instance **Figure 7**). The mentioned top-down modifications are then a process in which GAS modifies/increases the likelihood of PAM integration into SAS. Physiologically such a process is likely to involve modifications (e.g., partial disinhibition) of synaptic connections associating the individual EFs (and thereby PAMs) within SAS.

The constant interaction between GAS and SAS is essential for the content and form of mental representation as well as behavioral manifestations. Although individual examples such as those given above may leave the impression that GAS “dictates” mental representations and behavior, GAS is also highly influenced by SAS. The “goals” represented by GAS are the product of (partly environmentally determined) structures within SAS. And GAS represents “goals” in a broad sense which also included “predictions” regarding what is to be perceived and which behavioral patterns are to be performed. In these respects the REFGEN model agrees with the concept of predictive coding (e.g., Friston, 2005). Constantly interacting with SAS, GAS creates a configuration which – based on a combination of the current situation and prior experience – represents a “best prediction” regarding the perceptual situation as well as the most adequate behavioral and mental configurations. Such predictions are constantly “updated” – via the constant interaction between SAS and GAS. GAS can exert “top-down” influences on the mechanisms of perceptual receptiveness described by the REFCON model. Such influences may take the form of “attentional” processes favoring the integration of PAMs associated with for instance a particular part of the visual field. Or the influences may be a more specific “predictive coding” pre-activating PAMs associated with for instance a previously experienced image. In both cases, the perceptual process related to a given stimulus (including the potential conscious awareness of the stimulus) will be enhanced by the influences of GAS. According to the REFCON and REFGEN models, the degree of conscious experience of a stimulus is mediated by the degree of integration of PAMs into SAS. The PAMs in question include elements (EFs) associated with primary sensory areas (PEFs) and numerous other cortical and subcortical regions. Consequently, one should expect the consciousness-related neural processes as well as effects of predictive coding and influences of GAS to be found within both primary sensory areas and other brain regions. Regarding the time-course of electrophysiological influences this translates into both early and later effects. Recently (Andersen et al., 2016) it has been demonstrated that occipital electrophysiological activity with a latency of less than 300 ms predicts changes in the conscious perception of a visual stimulus. Aru et al. (2016) have found that prior experience with a visual stimulus affects both the conscious awareness of the stimulus and electrophysiological processes within a time window of 80–95 ms (with sources localized to occipital and posterior parietal cortical regions). Such results are in agreement with the predictions of the REFCON and REFGEN models.

According to predictive coding (e.g., Friston, 2005), one should expect prior experience with a given stimulus to be reflected in a reduced neural response when the stimulus is presented. As discussed by for instance Aru et al. (2016) prior presentation of a stimulus (and the consequent increased conscious detection of that stimulus) is not always accompanied by such a reduced neural response – occasionally the reverse (an increased neural activity) is found in such a situation. According to the REFGEN model such differences (decreased and increased neural response, respectively) primarily reflects the nature of the “expectancy” imposed by GAS on SAS. It may manifest itself as a general “readiness” of PAMs within for instance a given region of the visual field or some other more generalized dimension (in which case an increased neural activity would be expected) – such a situation represents traditional “attentional” processes. Or the influence may be a specific pre-activation of the pattern of PAMs associated with the presented stimulus (reflecting itself in a decreased neural response) – being a more specific “prediction.” Consequently, according to the REFGEN model the type of “predictive influences” emerging from (or via) GAS may take a form fully agreeing with predictive coding as described by Friston (2005). But it can also – depending on situational and to an extent individual factors – result in patterns of activation contrary to that kind of predictive coding.

The processes described by the REFGEN model – and its predictions – are best examined using methods able to detect rapidly changing patterns of activity. In animal models it may be possible to map the dynamically changing activation patterns associated with the recruitment and application of behavioral task solution strategies by the combined use of two-photon imaging and virtual spatial tasks (e.g., Stosiek et al., 2003; Harvey et al., 2012). In humans (where NCC may be more directly addressed) the GAS and SAS associated activation patterns may best be addressed (under various task/test conditions) by the analyses of EEG based microstates (e.g., Lehmann et al., 1987, 2006; Wackermann et al., 1993; Brandels et al., 1995; Pascual-Marqui et al., 1995; Cantero et al., 1999; Brier et al., 2010; Musso et al., 2010; Yuan et al., 2012; Cacioppo et al., 2015).

Many predictions of the REFGEN model will have to be examined – using such methods and others. Like other theories of NCC – e.g., IIT (e.g., Tononi et al., 2016) – REFGEN predicts a distributed but integrated NCC. Being based on the basic REF principles, however, the REFGEN model emphasizes that the relevant patterns of interconnectivity between EFs are highly dependent on experience. Consequently, the REFGEN model predicts that not only will the activity patterns (e.g., EEG based microstates) associated with consciousness differ between experimental conditions. They will also differ between individuals with different previous experiences. In within-subject designs this prediction can be tested by mapping the activation patterns associated with a given test situation (in which the conscious experience of the subject is evaluated via for instance PAS) before and after a specific training procedure addressing aspects of the relevant process (e.g., perception). The REFGEN model predicts a training associated reorganization of the SAS/GAS configuration – and consequently of the measured activity patterns. Such training effects should also be addressed using different types of feedback since the relevant reorganizations according to the model depend crucially on the provided feedback and consequent backpropagation processes.

Although many of the present examples of REFGEN related processes focus on neocortical mediation, subcortical mediation of elements within SAS, GAS, and various ASs should not be overlooked. In the process of selection of the AS to be activated as the basis for a given behavior or mental process, for instance, neostriatal mediation is likely to be involved in at least some situations. Such a mediation agrees well with the recently proposed model by Lisman (2014).

## The REF Complex – Toward A Framework for Understanding Connections and the NCC

The REFGEN model (as well as the REFCON and REF models) will be subjected to modifications and expansions as further data and theoretical developments indicate such adjustments. Presently, for instance, there is a need for the SAS of both the REFGEN and REFCON models to be split into two parallel entities. Conceptually there is a distinction between “first order” consciousness (perceptions, thoughts, and emotions) and the “second order” of metacognitive processes – including introspection (e.g., Metcalfe and Shimamura, 1994; Fleming and Frith, 2014). Such a distinction is supported by experimental indications that introspection and first order consciousness differ regarding cognitive (e.g., Stefanics et al., 2010; Wyart et al., 2012; Sherman et al., 2015) and neural (Guggisberg et al., 2011) processes. When attempting to model the relationship between first order consciousness and introspection (and more generally between primary cognition and metacognition) all previous models have been based on serial processes – either with or without an interactive component between first order consciousness and introspection (e.g., Dretske, 1995; Aru et al., 2012). Either primary consciousness has in such models been “read” by introspective processes or the introspective process has been seen as being able to modify the primary consciousness. Neither of these approaches is, however, compatible with all of the existing evidence (Overgaard and Mogensen, 2017). Attempting to create a model which can accommodate the available data regarding the differentiation and apparent interaction between primary consciousness and introspection we have proposed a new model, The Integrative Model (Overgaard and Mogensen, 2017). The Integrative Model presents primary consciousness and introspection-related processes as parallel processes representing gradual elaboration of representations. Although not explicitly a part of the REF framework, The Integrative Model takes as its offset the REF and REFCON models and it is to be implemented within both the REFCON and REFGEN models. Consequently, the presently described SAS of the REFGEN model will in such an elaboration consist of two parallel “layers”: SAS-A and SAS-B. While SAS-A is the basis of primary consciousness (and other primary cognitive processes) SAS-B mediates metacognitive processes – including introspection.

We believe that the REF complex – the original REF model, the REFCON model, and the REFGEN model – jointly constitutes a promising approach to the construction of a computational level bridging and integrating the neural level with the level of mental states/behavioral phenomena. Thereby the REF complex will also (as discussed in the “Introduction”) be able to provide a framework for the experimental approach to and interpretation of data from studies mapping the human connectome.

As importantly, this framework – and not the least the REFGEN model – conceptualizes consciousness and the NCC as an integral part of the complete cognitive system(s) of the individual (and of the neural substrates of these cognitive processes). As mentioned in the “Introduction,” we find such an integration to be a necessary consequence of the results pointing to consciousness being an aspect of in principle all cognitive processes – rather than an independent “module” (e.g., Overgaard and Mogensen, 2014, 2015, 2017).

Studies addressing the computational nature of EFs mediated by specific parts of the brain may be useful regarding the identification of meaningful selection and delineation of “nodes” (e.g., Sporns, 2013). The REF complex also emphasizes the relevance of considering the issues of individual variability, structural plasticity, and structure-function relationships [to use the terminology of Sporns (2013)]. In agreement with Woolrich and Stephan (2013) the REF complex stresses the importance of considering the structure-function relationships as well as the context-sensitive patterns that are modulated by task situations. The dynamic reorganizations described by the REF complex point to the need for analyzing the functional connectivity of the human brain under experimental circumstances specifically addressing both the short- and long-term modifications of connectivity. While comparisons of connectivity patterns under conditions of rest and functional activation (e.g., Smith et al., 2009; Deco et al., 2013) are important, it is crucial also to address functional connectivity as seen during a broad spectrum of stimulation and problem solving situations. Comparisons between individuals with various specific backgrounds should also be a focus of such research. Studies addressing sex differences (e.g., Ingalhalikar et al., 2014) are an important step which, however, needs to be supplemented by studies comparing same gender individuals with specific short- and long-term experiences – as, for instance, emphasized by the results of Sluming et al. (2007).

An understanding of the neurocognitive mechanisms within the REF complex may also provide a framework within which better to understand patterns of connectivity revealed by the studies of the human connectome. For instance, it will be interesting to integrate the REF related mechanisms with the findings of the “rich-club” organization in which a relatively small group of strongly interconnected hub regions were found to be more densely interconnected than what would have been predicted on basis of the average hub connectivity (van den Heuvel and Sporns, 2011).

And last, but not least, the REF framework provides an integrative approach within which consciousness is conceptualized as deeply integrated within other cognitive domains – and within which the growing understanding of the human connectome can better be utilized in the search for the NCC.

## Author Contributions

JM and MO contributed significantly to both the described theoretical developments and the writing of the present paper.

## Conflict of Interest Statement

The authors declare that the research was conducted in the absence of any commercial or financial relationships that could be construed as a potential conflict of interest.

## Abbreviations

AM: algorithmic module
AS: algorithmic strategy
EF: elementary function
GAS: goal algorithmic strategy
NCC: neural correlate of consciousness
PAM: perceptual algorithmic module
PAS: perceptual awareness scale
PEF: perceptual elementary function
REF: reorganization of elementary functions
REFCON: reorganization of elementary functions and consciousness
REFGEN: general reorganization of elementary functions
SAS: situational algorithmic strategy
